# Metabolic characteristics of individuals at a high risk of type 2 diabetes – a comparative cross-sectional study

**DOI:** 10.1186/s12902-017-0191-5

**Published:** 2017-07-14

**Authors:** Josefin Henninger, Araz Rawshani, Ann Hammarstedt, Björn Eliasson

**Affiliations:** The Lundberg Laboratory for Diabetes Research, Department of Molecular and Clinical Medicine, the Sahlgrenska Academy at the University of Gothenburg, 413 45 Gothenburg, Sweden

**Keywords:** Type 2 diabetes, First-degree relatives, Cross-sectional study

## Abstract

**Background:**

Type 2 diabetes (T2D) is associated with substantial morbidity and mortality. Individuals with a family history of T2D are at an increased risk of developing the disease. The aim of this study was to assess metabolic differences between first-degree relatives (FDR) of T2D patients and persons with no known family history of T2D (non-FDR).

**Methods:**

In 200 FDR and 73 non-FDR, we compared anthropometrics, glucose tolerance status, different measurements of insulin secretion, insulin resistance, as well as blood lipids and other blood analyses.

**Results:**

In the FDR group, 30 individuals had impaired glucose tolerance or T2D. Among the non-FDR, two individuals had impaired glucose tolerance. In unadjusted data, the FDR were older, had stronger heredity for coronary heart disease, lower body mass index and weight, higher OGTT plasma glucose concentrations, and impaired insulin secretion (all *p* < 0.05). Using propensity score, we matched the groups, resulting in significantly stronger heredity of coronary heart disease, higher OGTT plasma glucose at 60 and 90 min, larger glucose area under curve during the OGTT and higher serum creatinine among the FDR. Using least squares means, OGTT glucose at 60 and 120 min, as well as the area under curve, and OGTT insulin levels at 60 min were significantly higher. Body mass index was negatively correlated with insulin sensitivity (MI) and positively correlated with HOMA-β, a measurement of insulin secretion.

**Conclusions:**

We show that FDR are more likely to have impaired glucose tolerance and display higher OGTT plasma glucose and insulin, indicating an unfavorable metabolic profile. We conclude that OGTT is a simple and yet informative metabolic assessment in the FDR group. In both groups, we saw a negative correlation between body mass index and MI, confirming the role of body mass index in insulin resistance.

## Background

Type 2 diabetes mellitus (T2D) is a heterogeneous disease characterized by chronically elevated plasma glucose levels, as well as disturbances in the metabolism of lipids, carbohydrates and protein [1, 2]. It is intimately associated with obesity and the metabolic syndrome [2–4], which are major risk factors of cardiovascular disease [5], the number one cause of death globally today [4, 6].

The pathophysiology behind T2D has been extensively studied and there is consensus in the research community today that the pathophysiology indeed is multifactorial [1, 2, 7]. DeFronzo et al. summarizes how consensus has grown from acknowledging functional disturbances in pancreatic islet β-cell (ie insulin secretion) and the liver and muscle cells (ie insulin action), to today include well documented perturbations in adipose tissue metabolism and morphology, gut hormone regulation (ie incretin action), kidney function and pancreatic α-cell function, as well as central nervous system neurotransmitter dysfunction [1].

Lifestyle factors, in particular a hypercaloric diet, cigarette smoking and a sedentary lifestyle, in combination with genetic factors, are considered central underlying causes [8, 9]. In spite of only weak associations between specific genes and the development of T2D, first-degree relatives (FDR) of T2D patients are clearly at an elevated risk of developing the same disease, proportionate to the number of family members affected [2, 3, 10, 11].

Previous studies have shown that FDR display unfavorable anthropometrics and metabolic characteristics in spite of normoglycemia, however less refined methods have been used [12–17]. The aim of this study is to assess anthropometric and metabolic differences, using state of the art methods, between a group of healthy FDR and a group of healthy persons without known family history of diabetes, recruited from the background population.

## Methods

### Study populations

A total of 273 subjects were recruited between 2003 and 2014. Two hundred subjects were FDR and 73 were persons with no known family history of T2D (non-FDR). Additional inclusion criteria were no diabetes according to fasting plasma glucose, subjectively good health and no ongoing medications. The subjects were recruited through advertisements in local newspapers and public areas throughout the Gothenburg urban area.

### Variables

All tests were performed after a fast of a minimum of 12 h. At a first visit, we collected anthropometric data. Body weight and height, and waist and hip circumferences were measured manually by a research nurse using a weighing scale that was calibrated on several occasions during the study, and a measuring tape, BMI was calculated, and the proportions of body fat and lean body mass (LBM) were determined using bioelectrical impedance (single frequency, 50 kHz; Animeter, HTS, Odense, Denmark). Blood pressure was measured in a sitting position after a five min rest with a mercury sphygmomanometer. A venous blood sample of 10 mL was also taken for biochemical analyses. These were analyzed at the accredited central laboratory (Department for Clinical Chemistry, Sahlgrenska University Hospital, Gothenburg, Sweden) according to local standards. Total serum cholesterol, serum HDL cholesterol, triglycerides, ALT, ALP and serum creatinine, as well as plasma glucose were analyzed using photometry. All other biochemical analyses were performed by enzyme-linked immunoassay (ELISA) technique. Serum insulin was analyzed at the Wallenberg laboratory using a radioimmunoassay technique (Pharmacia, Uppsala, Sweden). Plasma insulin was measured at the University of Tübingen, Germany, by micro-particle enzyme immunoassay (Abbott Laboratories, Tokyo, Japan). A conversion factor was used to compare the two. Serum adiponectin was measured by a human adiponectin ELISA-kit (B-Bridge International, Sunnyville, CA, USA). Serum LDL cholesterol was calculated using the Friedewald equation [18]. At this visit the subjects also filled out a questionnaire regarding lifestyle and family history of diabetes. High heredity for T2D was defined as having more than one FDR with the disease. Level of stress was subjectively assessed.

At a second visit, an OGTT was performed. We used the WHO criteria for IGT and T2D [19]. The OGTT was performed by oral administration of 75 g of liquidized glucose at a fasting state and subsequent intravenous blood glucose samples (in mmol/L) at 30, 60, 90 and 120 min. The 120 min-value was used to assess glucose tolerance status as NGT, IGT or T2D [20]. To further assess glucose tolerance status, the concentrations of glucose and insulin concentrations were plotted over time. An area under curve (AUC) of glucose and insulin was calculated using the trapezoid method [21].

We also analyzed serum (in the non-FDR group) and plasma (in the FDR group) insulin levels (pmol/L) at 0, 30, 60, 90 and 120 min. HOMA-β was calculated using fasting glucose and fasting insulin levels [22]. The formula looks as follows: $HOMA- beta\%=\frac{fasting\  plasma\  insulin\ \left(\frac{pmol}{L}\right)\times 20\div 6.945}{fasting\  plasma\  glucose\ \left(\frac{mmol}{L}\right)-3.5}$

IGI (pmol/mmol) was calculated using the following formula: $\frac{\varDelta\ plasma\  insulin\kern0.75em \left(\frac{pmol}{L}\right)}{\varDelta\ plasma\  glucose\kern0.75em \left(\frac{mmol}{L}\right)}$

The delta values are the difference between the plasma values at 120 min and fasting values during the OGTT [23, 24].

At the third visit, a hyperinsulinemic, euglycemic clamp - the gold standard and reference method to assess insulin sensitivity - was performed on each subject. A disposition index, derived from IGI from the OGTT and the M/I-value from the clamp performed at this visit, was also calculated [25], thus providing a β-cell function estimate superior to the IGI and the HOMA-beta, as it takes insulin resistance into account [26]: $$ \frac{ I GI}{\frac{1}{\raisebox{1ex}{$ M$}\!\left/ \!\raisebox{-1ex}{$ I$}\right.}}= IGI\kern0.5em \times \left(\frac{M}{I}\right)=\frac{pmol}{mmol}\times \frac{mg\div kg\div \mathit{\min}}{pmol\div L}=\frac{mL}{mol\div \mathit{\min}} $$

The clamp was performed in a fasting state by a continuous intravenous infusion of insulin, to suppress endogenous insulin production, and subsequent and variable glucose infusion. Glucose was measured at five min intervals until a steady state was reached, where plasma glucose was clamped at 5.0 mmol/L. At the clamped plasma glucose level, the glucose infusion rate (GIR, ie M-value), divided by lean body mass was calculated as a measurement of the whole body glucose uptake [27]. Also, to account for endogenous insulin levels, an M/I (I being insulin levels during the steady-state phase of the clamp) was also calculated [28].

### Statistical analysis

Analyses were performed using SPSS statistics version 22.0 (Armonk, NY, USA: IBM Corp.), as well as R (The R Project for Statistical Computing version 3.1.2). Variables were considered normally distributed after visual assessment in a histogram. The results for continuous variables are given as means and SD, and for categorical variables as frequencies. Student’s t-test was used to assess statistical significance between groups for continuous variables and for categorical variables Chi-square test was used. A value of *p* < 0.05 was considered statistically significant. However, the *p* values should be interpreted in the light of the exploratory nature of the study and the number of tests performed.

We used linear regression to compare IGI, HOMA-β, DI, M, MI and plasma insulin and glucose, as well as insulin and glucose AUC, between non-FDR and FDR. Adjustment was made for age, sex, BMI, physical exercise and smoking. To obtain a mean value for each dependent variable, we used least squares means with the same model specifications. Group differences were examined with and without imputation of missing data. Missing data was imputed using the MICE algorithm (we imputed 5 complete data sets).

We also performed a 1-to-1 matching, using a propensity score. We matched each non-FDR subject to one FDR subject, conditional on age, sex, and BMI. The resulting matched group consisted of 146 individuals; 73 subjects in each group. We compared the groups directly in order to explore metabolic differences between non-FDR and FDR at the same age, sex and BMI.

Correlations between BMI and fat percent on one hand, and IGI, MI and HOMA-β on the other in non-FDR and FDR were also assessed, by use of Spearman’s correlation [29–31].

## Results

Two hundred subjects were FDR and 73 were non-FDR. In the FDR group, 170 individuals displayed NGT, 29 had IGT, 6 had IFG (of which 3 also had IGT) and 1 individual fulfilled the T2D criteria. Among the non-FDR, 70 were NGT, 2 individuals had IGT and 1 had IFG.

### Crude characteristics

Clinical, anthropometric and biochemical characteristics of the complete and unmatched two groups are displayed in Table 1. The FDR were significantly older, displayed stronger heredity for CHD, and had lower BMI and weight, but still similar WHR supporting an increased predisposition for an abdominal adipose tissue profile. They also displayed higher plasma glucose concentrations during the OGTT, including AUC, however at equal insulin concentrations, as well as higher serum HDL and lower LDL. There were non-significant differences in insulin sensitivity (*p*-values <0.1), while measurements of insulin secretion showed that the FDR had lower HOMA-β, higher IGI and DI were not statistically significantly different.

**Table 1:** Subject characteristics of the complete two groups, unadjusted

	Non-FDR	FDR	p
N	73	200	
Sex male (%)	33 (45.2)	81 (40.5)	0.576
Age (years)	36.68 (7.99)	40.05 (7.16)	0.001
Weight (kg)	83.93 (14.86)	75.98 (12.95)	<0.001
BMI (kg/m^2^)	27.22 (3.91)	25.14 (3.47)	<0.001
Fat percent (%)	27.87 (9.24)	26.42 (8.09)	0.218
DI (mL/mol/min)	3.63 (3.56)	2.78 (2.88)	0.108
Waist circumference (cm)	90.37 (10.64)	88.39 (10.20)	0.169
WHR	0.85 (0.08)	0.86 (0.08)	0.334
Diastolic blood pressure (mmHg)	77.53 (8.98)	73.64 (9.75)	0.003
Systolic blood pressure (mmHg)	121.19 (14.50)	115.99 (12.22)	0.004
High heredity for T2D (%)	0 (0.0)	7 (3.6)	<0.001
High heredity for CHD (%)	9 (13.0)	76 (29.6)	0.001
Currently smoking (%)	7 (10.2)	22 (11.3)	0.877
Exercise >4 times per week (%)	41 (57.7)	68 (35.2)	0.008
High stress level (%)	9 (13.0)	34 (17.7)	0.010
Serum adiponectin (ng/mL)	8295.29 (3551.68)	9064.92 (4436.85)	0.233
Serum triglycerides (mmol/L)	0.91 (0.48)	1.03 (0.54)	0.131
Serum HDL (mmol/L)	1.42 (0.32)	1.55 (0.38)	0.031
Serum cholesterol (mmol/L)	4.66 (0.86)	4.82 (0.89)	0.185
Serum LDL (mmol/L)	3.78 (0.80)	2.84 (0.84)	<0.001
Serum creatinine (μmol/L)	74.91 (12.45)	85.86 (16.81)	<0.001
OGTT fasting plasma glucose (mmol/L)	4.74 (0.36)	4.81 (0.42)	0.165
OGTT plasma glucose 30 min (mmol/L)	7.55 (1.54)	7.99 (1.54)	0.044
OGTT plasma glucose 60 min (mmol/L)	6.90 (1.90)	7.76 (2.12)	0.003
OGTT plasma glucose 90 min (mmol/L)	5.87 (1.51)	6.95 (1.89)	<0.001
OGTT plasma glucose 120 min (mmol/L)	5.56 (1.37)	6.12 (1.59)	0.010
OGTT fasting serum insulin (pmol/L)	58.77 (36.19)	49.20 (29.30)	0.048
OGTT serum insulin 30 min (pmol/L)	489.05 (336.05)	412.03 (245.01)	0.068
OGTT serum insulin 60 min (pmol/L)	454.58 (297.68)	431.35 (261.66)	0.572
OGTT serum insulin 90 min (pmol/L)	336.85 (257.46)	396.38 (297.55)	0.162
OGTT serum insulin 120 min (pmol/L)	305.38 (267.73)	318.93 (288.16)	0.746
HOMA-β (%)	150.53 (81.27)	118.88 (63.90)	0.003
IGI (pmol/mmol)	194.70 (250.13)	136.18 (144.01)	0.044
M-value (GIR/kg/min: g/kg/min)	11.94 (4.49)	13.00 (4.16)	0.085
M/I	0.02 (0.01)	0.02 (0.01)	0.083
OGTT insulin AUC (pmol/L/120 min	44,235.77 (28,021.78)	43,030.71 (25,137.08)	0.764
OGTT glucose AUC (mmol/L/120 min	763.75 (149.28)	844.66 (166.09)	<0.001
HOMA-IR	12.58 (2.27)	10.69 (0.19)	0.092

Data are, for categorical variables, given as frequencies (percentage) and for continuous variables as mean (standard deviation). *P*-values have been obtained as explained in the method section. *FDR* first-degree relatives, *WHR* waist-hip ratio, *T2D* type 2 diabetes, *CHD* coronary heart disease, *HDL* high-density lipoprotein, *LDL* low-density lipoprotein, *HOMA-β* homeostasis assessment model-beta, *IGI* insulinogenic index, *DI* disposition index, *M and M/I* insulin resistance level, *GIR* glucose infusion rate

### Differences and similarities in the matched set

Clinical, anthropometric and biochemical characteristics in the matched set, using propensity score, are displayed in Table 2. When comparing the two matched groups of individuals, FDR only had a significantly higher level of heredity for CHD, higher OGTT plasma glucose at 60 and 90 min, as well as AUC, lower serum LDL and higher serum creatinine.

**Table 2:** Subject characteristics after matching by propensity score

	Non-FDR	FDR	p
N	73	73	
Sex male (%)	33 (45.2)	35 (47.9)	0.868
Age (years)	36.68 (7.99)	35.99 (6.82)	0.571
Weight (kg)	83.93 (14.86)	81.83 (14.71)	0.391
BMI (kg/m^2^)	27.22 (3.91)	26.61 (4.14)	0.356
Fat percent (%)	27.87 (9.24)	27.18 (8.69)	0.647
DI (mL/mol/min)	3.63 (3.56)	2.52 (2.77)	0.085
Waist circumference (cm)	90.37 (10.64)	92.15 (11.59)	0.343
WHR	0.85 (0.08)	0.88 (0.09)	0.060
Diastolic blood pressure (mmHg)	77.53 (8.98)	74.68 (9.95)	0.073
Systolic blood pressure (mmHg)	121.19 (14.50)	115.84 (12.60)	0.019
High heredity for T2D (%)	0 (0.0)	3 (4.1)	<0.001
High heredity for CHD (%)	9 (13.0)	24 (32.9)	0.012
Currently smoking (%)	7 (10.0)	8 (11.0)	0.943
Exercise >4 times per week (%)	41 (57.7)	33 (45.2)	0.275
High stress level (%)	9 (13.0)	16 (21.9)	0.235
Serum adiponectin (ng/mL)	8295.29 (3551.68)	7525.09 (3707.54)	0.242
Serum triglycerides (mmol/L)	0.91 (0.48)	1.13 (0.68)	0.038
Serum HDL (mmol/L)	1.42 (0.32)	1.47 (0.36)	0.418
Serum cholesterol (mmol/L)	4.66 (0.86)	4.68 (0.89)	0.885
Serum LDL (mmol/L)	3.78 (0.80)	2.59 (0.67)	<0.001
Serum creatinine (μmol/L)	74.91 (12.45)	88.44 (15.75)	<0.001
OGTT fasting plasma glucose (mmol/L)	4.74 (0.36)	4.85 (0.49)	0.123
OGTT plasma glucose 30 min (mmol/L)	7.55 (1.54)	8.15 (1.39)	0.017
OGTT plasma glucose 60 min (mmol/L)	6.90 (1.90)	7.99 (2.24)	0.002
OGTT plasma glucose 90 min (mmol/L)	5.87 (1.51)	6.97 (1.99)	<0.001
OGTT plasma glucose 120 min (mmol/L)	5.56 (1.37)	6.11 (1.61)	0.032
OGTT fasting serum insulin (pmol/L)	58.77 (36.19)	57.98 (36.78)	0.906
OGTT serum insulin 30 min (pmol/L)	489.05 (336.05)	457.50 (274.58)	0.580
OGTT serum insulin 60 min (pmol/L)	454.58 (297.68)	498.54 (314.31)	0.429
OGTT serum insulin 90 min (pmol/L)	336.85 (257.46)	461.19 (371.26)	0.030
OGTT serum insulin 120 min (pmol/L)	305.38 (267.73)	401.78 (380.42)	0.103
HOMA-β (%)	150.53 (81.27)	132.58 (72.04)	0.209
IGI (pmol/mmol)	194.70 (250.13)	135.21 (103.17)	0.111
M-value (GIR/kg/min: g/kg/min)	11.94 (4.49)	11.87 (3.89)	0.927
M/I	0.02 (0.01)	0.02 (0.01)	0.816
OGTT insulin AUC (pmol/L/120 min	44,235.77 (28,021.78)	50,099.93 (30,732.69)	0.284
OGTT glucose AUC (mmol/L/120 min	763.75 (149.28)	857.29 (172.61)	0.001
HOMA-IR	12.58 (8.36)	12.81 (8.67)	0.879

Data are, for categorical variables, given as frequencies (percentage) and for continuous variables as mean (standard deviation). *P*-values have been obtained as explained in the method section. *FDR* first-degree relatives, *WHR* waist-hip ratio, *T2D* type 2 diabetes, *CHD* coronary heart disease, *HDL* high-density lipoprotein, *LDL* low-density lipoprotein, *HOMA-β* homeostasis assessment model-beta, *IGI* insulinogenic index, *DI* disposition index, *M and M/I* insulin resistance level, *GIR* glucose infusion rate

**Fig. 1:**
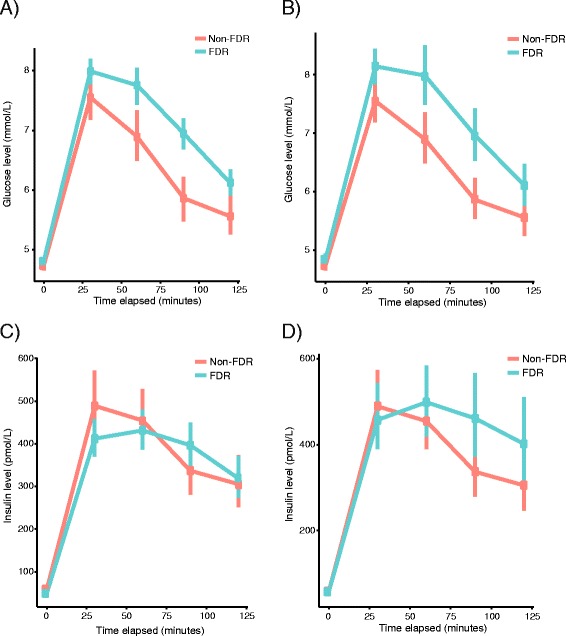
**a** OGTT plasma glucose response in the overall set. OGTT = oral glucose tolerance test. AUC = area under curve. **b** OGTT plasma glucose response in the matched set (*n* = 146). OGTT = oral glucose tolerance test. AUC = area under curve. **c** OGTT plasma insulin response in the overall set. OGTT = oral glucose tolerance test. AUC = area under curve. **d** OGTT plasma insulin response in the matched set (*n* = 146). OGTT = oral glucose tolerance test. AUC = area under curve

Figure 1 a-d show mean plasma glucose and insulin concentrations during the OGTT in non-FDR and FDR, overall and in the matched set. Elevations in plasma concentration of glucose can be seen throughout the trial, including a larger AUC in the FDR group. AUC for plasma insulin did not differ significantly between groups.

We examined least square means and 95% confidence intervals for IGI, HOMA-β, DI, M, MI and OGTT glucose and insulin, including AUC. Means and confidence intervals were derived from the complete cases, whereas *p* values were pooled over the 5 imputed data sets. OGTT glucose at 60 and 120 min, as well as AUC glucose were significantly higher, and OGTT insulin at 60 min were significantly higher among FDR (*p* < 0.05). The other examined variables did not reach statistical significance, however insulin and glucose levels tended to be higher in the FDR group throughout the OGTT. Also, IGI and DI both tended to be lower in the FDR group, indicating impaired β-cell function. These figure have not been included in this publication.

### Correlations

**Fig. 2:**
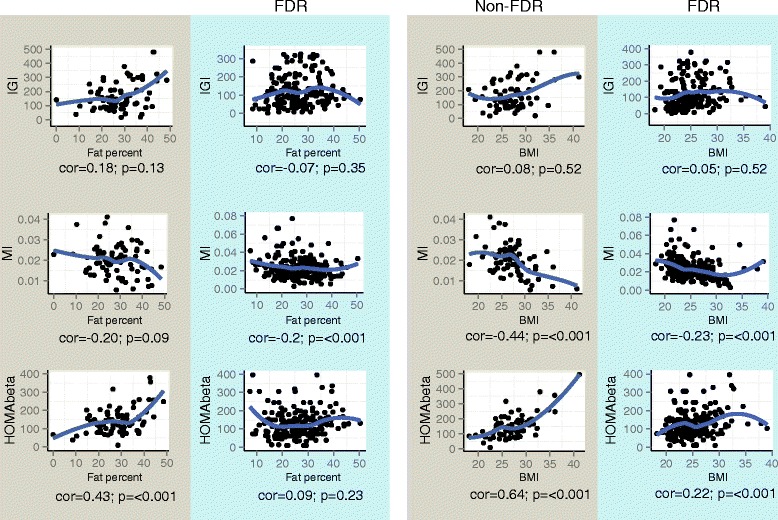
Correlation between measurements of insulin secretion and insulin resistance, and fat percent and BMI, calculated as described in Methods. BMI = body mass index. IGI = insulinogenic index. MI = insulin resistance level

We examined correlations between BMI and fat percent, and IGI, MI, HOMA-β in both groups, including all individuals (Fig. 2). In neither of the groups we saw a statistical significant correlation between IGI and BMI or fat percent. BMI was negatively correlated with MI and positively correlated with HOMA-β in both non-FDR and FDR, although more strongly in non-FDR. When examining which correlations were present with fat percentage, only a relatively weak negative correlation with MI in the FDR group and a positive correlation with HOMA-β among non-FDR reached statistical significance.

## Discussion

In summary, we found that healthy FDR display higher glucose and insulin levels during an OGTT, higherserum triglycerides and a tendency towards disturbed β-cell dysfunction, when compared to subjects without a family history of diabetes. Using covariate analysis, we also found that OGTT glucose at 60 and 120 min, aswell as AUC glucose were significantly higher, and OGTT insulin at 60 min were significantly higher, among FDR. We also found that in the FDR group, fat percentage and BMI were both negatively correlated with MI.

A primary finding of this study was the elevated insulin and glucose levels during and OGTT, despite equal insulin resistance levels between groups, and thus confirming the OGTT usefulness in detecting high-risk individuals, despite its simplicity. Our data, both adjusted and unadjusted, showed higher plasma glucose and insulin concentrations, including AUC and iAUC, throughout the OGTT in the FDR compared to non-FDR. Other studies have thoroughly examined OGTT findings in FDR subjects, and have reached similar conclusions. Taheri et al. and Alyass et al. hypothesized, that measurements at 60 min during an OGTT might detect disturbances not shown at 120 min [10, 12, 13, 32, 33]. In the Baltimore Longitudinal study of ageing, OGTT data, including AUC, from 1400 non-diabetic individuals showed that integrated glucose, ie glucose AUC, as well as glucose concentration at 120 min, were significant predictors of all-cause mortality [34].

In this study, we also aimed to study insulin secretion function and found that there is a tendency, however not statistically significant, in the FDR group, towards β-cell dysfunction, compared to the non-FDR. In our matched results, one could also see a tendency in insulin concentrations from the OGTT, that FDR had higher fasting insulin levels, lower 30 min values and then higher concentrations at 60, 90 and 120 min. Possibly somewhat speculative, this further indicates β-cell dysfunction with a slow acute insulin response and a compensatory overproduction during the late response. In the previously mentioned study by Stadler et al., disturbances in insulin secretion (IGI) were also observed in the FDR group. They showed that acute phase insulin secretion was diminished and hyperinsulinemia during the late phase was present, despite normal insulin sensitivity [10]. Perseghin et al. showed that when subdividing the FDR into groups according to M-value, the most insulin resistant individuals had a significantly lower acute insulin response than the normally insulin sensitive FDR, implying that the IR and β-cell dysfunction are indeed intimately connected, and more so than mere heredity [35]. Praveen et al. found that DI was lower in the FDR group than non-FDR, indicating β-cell dysfunction [13]. Gautier et al. concluded that FDR display early insulin secretion dysfunction during an IVGTT, despite normoglycemia [36]. Cnop et al. concluded that FDR’s decline in glucose tolerance status is mostly due to a loss in insulin secretion ability [37].

Increasing BMI and fat percentage have often been associated with increasing insulin resistance. We confirmed these correlations in our study population, and found that in both groups, there was indeed a negative correlation between BMI and insulin sensitivity. In both groups, increasing BMI was also associated with increasing HOMA-β, indicating a responding increase in insulin demand upon weight gain. However, the correlation coefficient was markedly lower among FDR than non-FDR, possibly indicating an inability to increase insulin production upon weight gain in the FDR group. Interestingly, we also found that among FDR, but not among non-FDR, increasing fat percentage was associated with decreasing MI, indicating an inability among the FDR to stay insulin sensitive upon expansion of the adipose tissue, specifically.

In our study, no differences in insulin resistance levels were seen, neither in adjusted nor unadjusted data. We hypothesize that this finding is possibly due to baseline differences between FDR and non-FDR, where the former had lower BMI. Other studies have shown both clamp data, M and MI, and surrogate measures such as HOMA-IR to be deranges in the FDR group. Stadler et al., as well as Nyholm et al., showed that signs of IR (lower M-value and M/I) were present in FDR when compared to a matched group of non-FDR [10, 32]. Praveen et al., as well as Kuzhandai et al. and Arner et al., displayed higher HOMA-IR, ie lower insulin sensitivity, in the FDR group than in non-FDR subjects [13, 14, 38]. Previous studies from our laboratory, performed on minor subgroups from the same study population but with more narrow inclusion criteria, have shown lower M and/or M/I values, as well as higher HOMA-IR and hypertriglyceridemia [38–40].

A major strength of this study was our usage of state of the art methodology to assess insulin sensitivity, as well as extensive measurements to assess β-cell function and adipose tissue function and biochemical data, including OGTT to assess glucose tolerance status and doing so in a relatively large study population [23, 27]. Other studies have looked at similar questions in similar study populations, however using less refined methodology [12–16].

Certain limitations to this study need to be addressed. In our results the non-FDR tended to be – not at a statistically significant level, however - more physically active, which could possibly have influenced our results. Also, we did not perform an IVGTT to assess β-cell function. In this study, we have not explored subjects’ dietary habits. Information on diet could have contributed to increase the scientific value of our results, as a hyper-caloric diet is a well-established contributing factor in the development of obesity and T2D. Specific studies on the FDR group have also concluded that unhealthy dietary habits contributed to FDR’s increased risk of cardiometabolic disease [2, 6, 41]. We did not include data on ethnicity, a known risk factor for glucose tolerance variations, in the two study groups. However, the vast majority of study participants were of Caucasian ethnicity, and we would thus not have had power to address if ethnicity was a contributing factor to differences between groups. Finally, among both groups were individuals with glucose tolerance disturbances, including one individual with overt T2D. However, the subjects were not included because they were normoglycemic, but because they were without known T2D and in general good health. We therefore have not performed any statistical analyses on subjects stratified by glucose tolerance status, as that in our opinion would make our data less representative of the background population. We consider it important to look at a population without too many modifications and excluding criteria, as that would make our sample less representative of the diverse background population.

Before matching, the FDR had lower mean BMI and less atherogenic blood lipid characteristics than the non-FDR. These differences in characteristics we believe were due to the non-FDR’s higher weight, BMI and WHR, despite lower age. Mode of recruitment could have produced this bias, as we hypothesized that the FDR were aware of their higher risk of developing T2D, and thus more prone to be motivated to both participate in the study, despite normal weight, and possibly also to stay leaner and lead a healthier lifestyle. This could be considered a selection bias on our part, and a methodological imperfection. Smaller studies on subgroups from our study population, such as Axelsen et al. and Johanson et al., around 15 cases, plus controls, were recruited and then one can find subjects at matching BMI’s [39, 42]. In our populations, with almost 300 subjects, seeing leaner FDR and heavier non-FDR was to be expected. However, despite being significantly leaner, the FDR were less glucose tolerant, adjusted both by matching and least squares means, indicating a more unfavorable metabolic profile despite lower BMI than non-FDR. Furthermore, the non-FDR had a slightly lower WHR, not reaching statistical significance, possibly indicating a slightly more favorable regional adiposity. These results confirmed what Cederberg et al. hypothesized, that FDR are at an increased risk of T2D, obesity and specifically obesity-related insulin resistance [43]. As this study has included the recruited groups in their totality, taken from the background population and using minimal exclusion criteria, avoiding bias and confounders would be very difficult. We consider it important to include broadly from a background population to explore correlations and tendencies, as an important complement to longitudinal studies, and randomized clinical trials in particular, from which causative factors can be determined.

## Conclusion

In this cross-sectional study of 200 FDR of T2D patients and 73 subjects without family history of T2D, we found that the FDR display glucose tolerance disturbances when compared to non-FDR, rather than insulin resistance. We also conclude that OGTT is a solid and informative, yet simple and inexpensive, mode of assessing risk of T2D in this high-risk group of FDR.

## Abbreviations

AIR: Acute insulin response
ALP: Alkaline phosphatase
ALT: Alanine aminotransferase
AUC: Area under curve
BMI: Body mass index (kg/m^2^)
CHD: Coronary heart disease
Clamp: Euglycemic, hyperinsulinemic clamp
DI: Disposition index
FDR: First-degree relative
FFA: Free fatty acid
HDL: High-density lipoprotein
HOMA-β: Homeostasis model assessment of the β-cell function index
IFG: Impaired fasting glucose
IGI: Insulinogenic index
IGT: Impaired glucose tolerance
IR: Insulin resistance
IVGTT: Intravenous glucose tolerance test
LDL: Low-density lipoprotein
LIR: Late insulin response
M (M-value): Glucose infusion rate/lean body mass during the euglycemic, hyperinsulinemic clamp
MI: M-value/insulin concentration during steady state of the euglycemic, hyperinsulinemic clamp
NGT: Normal glucose tolerance
OGTT: Oral glucose tolerance test
T2D: Type 2 diabetes mellitus
WHO: World health organization
WHR: Waist/hip-ratio
